# Enhancing computational thinking through coding education in primary school students: an experimental study on the impact of early programming exposure on problem-solving skills

**DOI:** 10.3389/fpsyg.2026.1734482

**Published:** 2026-02-18

**Authors:** Xin Wang, Feixue Wan, JiaMin Dai

**Affiliations:** 1 School of Computer Science and Technology/School of Artificial Intelligence, China University of Mining and Technology, Xuzhou, China; 2 Institute of Education/Center for Teaching and Learning Development, Xiamen University, Xiamen, China; 3 College of Statistics and Data Science, Xinjiang University of Finance & Economics, Xinjiang, China

**Keywords:** computational thinking, early coding exposure, educational intervention, problem-solving skills, scratch

## Abstract

**Introduction:**

This research paper presents an empirical investigation of the effectiveness of early coding instruction in improving problem-solving skills and computational thinking (CT) among primary school students. The primary research question was to determine whether a structured six-month coding intervention yields greater cognitive gains in children aged 8–12 than instruction without coding.

**Methods:**

The study employed a quasi-experimental pre-test/post-test design with 200 students randomly assigned to an experimental group (*n* = 100) and a control group (*n* = 100). The experimental group participated in a 24-week curriculum using Scratch and Python, while the control group followed the standard school curriculum. To assess skill acquisition and practice intensity, paired-sample *t*-tests, independent-sample *t*-tests, and Pearson correlation analyses were conducted.

**Results:**

The findings indicate statistically significant effects for the experimental group, with problem-solving scores increasing to 23.5 from a mean of 17.8 (*p* < 0.001) and computational thinking scores increasing to 30.6 from a mean of 20.4 (*p* < 0.001), reflecting large effect sizes. In contrast, the control group exhibited no significant gains.

**Conclusion:**

From a theoretical perspective, the study contributes to the literature by demonstrating that the concrete operational stage of development (ages 7–11) represents a critical period for the development of abstract algorithmic thinking through programming. From a practical standpoint, the results provide evidence-based support for integrating coding into primary education not merely as a professional competency, but as a cognitive development opportunity essential for 21st-century digital literacy.

## 1 Introduction

The computational thinker skill has now been used to cross boundaries and be a basic skill needed to fully participate in a digital society because of the fast changing environment of the 21st century. Computational thinking (CT) which is defined as thought processes that are used to formulate problems along with their solution in a form that can be effectively implemented by an information-processing agent (Ye et al., 2023) is now considered a critical literacy along with reading, writing and mathematics. The concept of coding and computer science is getting integrated into the K-12 curricula of governments and institutions worldwide, which is justified by the assumption that learning how to code leads to the enhancement of the cognitive domain (Şat and Kadırhan, 2024). Nevertheless, in spite of the fact that the theoretical grounds of early coding education are very strong, the empirical situation is undeveloped, especially when it comes to the rigorous experimental validation of these arguments in the case of primary school children between the age of 8 and 12.

To the best of our knowledge, there is a large research gap in the literature despite the enthusiasm of coding to all. Most of the available literature on coding education is based on the secondary or higher education students as their cognitive stages of development are not similar to the elementary learner (Mills et al., 2024). Moreover, most of the studies done on the primary level have been based on quasi-experimental models, small samples, or observational case-studies, which do not have the statistical strength to provide causal associations (Anuar and Ismail, 2025). Efficiency In converting young learners out of block-based systems such as Scratch to text-based systems such as Python within a single intervention period has not been studied extensively as well. This is a crucial gap since the cognitive development stage of the concrete operational stage (in most cases, 7–11 years old) is the one that is believed to be an ideal time to introduce logical structures and algorithmic reasoning (Jeon and Kwon, 2024). In the absence of strong experimental evidence, teachers and educators have no clear indication of how best to introduce coding interventions, when, and how much time they should take.

This paper will mitigate these shortcomings by using a randomized controlled trial (RCT) to conduct a rigorous investigative intervention of the effect of a 6-month coding intervention on 200 students in a primary school. This study aims to overcome the issue of anecdotal evidence by controlling confounding factors, as well as offering real facts in the area of skill acquisition due to its large sample. A research questions guiding the study is as follows:

*RQ1*: Does a structured coding education have a significant effect in enhancing the skills of computational thinking (decomposition, abstraction, pattern recognition, algorithmic thinking) among primary students as compared to a control group?

*RQ2*: How is the overall problem-solving skills of students not within a programming setting affected by early exposure to coding?

*RQ3*: What is the association between the cumulative number of coding hours and the size of the skill development?

The conceptual framework that is informing this study is the theory of constructionism formulated by Seymour Papert which holds that learning is best achieved when the learner engages him/herself in the process of constructing a significant artifact (Selamat et al., 2024). When it comes to coding, the students do not just receive the information; they externalize their thought processes and create games, stories, and animations. This outwardness can be debugged not only code but their own mental models, which in theory can result in better metacognition and problem-solving abilities (Tan et al., 2023). Moreover, the research relies on the definition of CT as a multidimensional construct that contains certain habits of cognition which can be developed with the help of the practice (Montuori et al., 2023).

The relevance of this research is due to its high standards of methodology and the importance of a critical age group. This study can be of assistance in the current debate on the cognitive benefits of computer science education by offering empirical data on how coding skills can be generalized to general problem-solving problems. It questions the belief that coding is a strictly vocational skill, and instead, it is presented as a conceptual underpinning of the contemporary intellectual growth. This exploration, as opposed to the current studies that could have depended on the general educational analytics, is a direct and measurable student outcomes based on controlled experimental environment.

## 2 Related works

### 2.1 Computational thinking frameworks in education

Being introduced to educational contexts in a number of ways, since being put forward by influential Wing, with the term under consideration, computational thinking has been put into operationalization. A framework of Scratch environment proposed by Rich and Browning (2019) suggests the classification of CT: concepts (e.g., loops, parallelism), practices (e.g., testing, debugging), and perspectives (e.g., expressing oneself). This framework has come to be a pillar in the evaluation of CT in the primary school. Recent research has placed more focus on the unplugged elements of CT and has proposed that decomposition and abstraction cognitive capacity can be instructed without the use of computers (Chen et al., 2023). Nonetheless, it is increasingly recognized that the iterative feedback loop that is supplied by real programming environments offers a scaffolding of such skills unplugged activities cannot deliver (Lye and Koh, 2014).

### 2.2 Coding education for young learners

Block-based programming languages have been largely used in research on coding elementary students. Experiments with Scratch have always reported a positive interaction and selfefficacy effect (Belessova et al., 2024). As an example, a quasi-experimental design by functionality showed that fourth-grade students were able to acquire complicated skills such as conditionals and variables presented in visual blocks effectively (Stolpe and Hällström, 2023). The shift towards text languages is however a challenge. Literature proposes that, although block-based coding makes the learning process easier, it might not equip students with the syntactic rigidity of professional languages (Al-Imamy, 2024). There is little research on curriculum designs in the intentional bridging of this gap in one academic year with this age group.

### 2.3 Project-based learning (PBL) and constructionism

Project-Based Learning (PBL) has close pedagogical value with the constructionist theory. PBL learning involves active participation of students in the real world and personally relevant projects. PBL reviews in STEM education show that PBL is an effective approach in terms of promoting student motivation and retention of content over conventional rote education (Krajcik and Blumenfeld, 2005). PBL in coding education changes the learning process where one memorizes syntax to the one where one expresses creatively. According to recent meta-analyses of STEM interventions, the active learning element of PBL can be considered one of the main moderators of effect size in education outcomes (Wijnia et al., 2024). The proposed study will take a PBL approach to make sure the proposed intervention is pedagogically sound and interest young learners.

### 2.4 Assessment of computational thinking

One of the most difficult aspects of the practice is the measurement of CT. Initial efforts were based on the analysis of the code: the numbers of blocks employed in a project. Yet, the process of thinking cannot be observed by such approach. Therefore, researchers have generated validated rubrics and psychometric measures that evaluate CT by using logic puzzles and scenario-based questions unrelated to particular coding syntax (Orhan et al., 2025). The credibility of these tools is imperative to prove that CT is a particular cognitive skill and not expertise in a particular software. According to recent systematic reviews of CT assessment instruments used in primary schools, it is necessary to have multidimensional instruments to measure the particular sub-skills such as an algorithmic thinking and pattern recognition separately (Orhan et al., 2025).

### 2.5 Synthesis and research gap

Although the literature determines the potential of coding in developing CT, research on the topic of primary school students in longitudinal studies or over time experiments is limited. With the current literature, the majority of studies are short-term (e.g., Hour of Code or oneweek camp) and, thus, not enough to trace profound changes in cognition (Anuar and Ismail, 2025). Moreover, the particular effect of the shift in the visual to text-based coding on the transfer of problem-solving is rather theoretical. These gaps are bridged in this study by using a six-month, intensive experimental intervention applying specifically the measurement of transfer of coding skills to general problem-solving domains based on validated measures.

## 3 Hypothesis

*Null Hypothesis (H0)*:

Early coding exposure does not have any significant effects on computation thinking and problem-solving skills.

*H0-1*: There is no significant difference between students who get coding instruction and students who fail to get coding instruction on their scores in computer thinking.

*H0-2*: There is no significant difference in the performance of the two groups in solving tasks.

*Alternative Hypothesis (H1)*:

Early exposure to programming enhances CT and problem solving skills of students.

*H1-1*: Students who receive coding teaching will start the test at least an order of magnitude higher in computational thinking than students who do not.

*H1-2*: The exposure of students to coding training will result in a significant improvement in their scores on problem-solving tasks as compared to students who did not receive this type of training.

## 4 Methods

### 4.1 Research design

The research design used in this study was a quantitative, true experimental research design which used a pre-test/ post-test control group design. The reason why the experimental design was selected is to develop a causal relationship between the independent variable (coding education intervention) and dependent variables (computational thinking skills and problem-solving abilities) (Creswell and Creswell, 2018). Through random distribution of subjects to groups, the design will eliminate internal validity threats of selection bias and maturation and will be able to ensure that any observed differences can be attributed to the intervention with a high level of certainty.

### 4.2 Participants

The population of the study was the students of three urban elementary schools that belong to the urban district and are based in primary school. Two hundred students (*N* = 200) between the ages of 8 and 12 years (Grades 36) took part in the research. The sample was even regarding the gender (48% female, 52% male) and reflected a wide range of socioeconomic background that is common in the region. Criteria of inclusion included the students not being trained in computer programming before in any formal way except normal school exposure. Participation was voluntary, and informed consent was taken by parents or legal guardians and student assent was taken in compliance with the ethical standards that were endorsed by institutional review board (IRB).

### 4.3 Group assignment

The randomly selected participants at two groups were allocated at random with the help of a computer-generated randomization sequence in order to maintain allocation concealment. Experimental Group (*n* = 100): In this group, the structured coding education intervention was applied and the Control Group (*n* = 100) maintained the regular school curriculum, including basic ICT literacy (e.g., typing, word processors) but not any deliberate instruction in coding or algorithms. Pre-test comparisons were taken to verify that there were no significant differences at the baseline of the groups.

**Figure 1:**
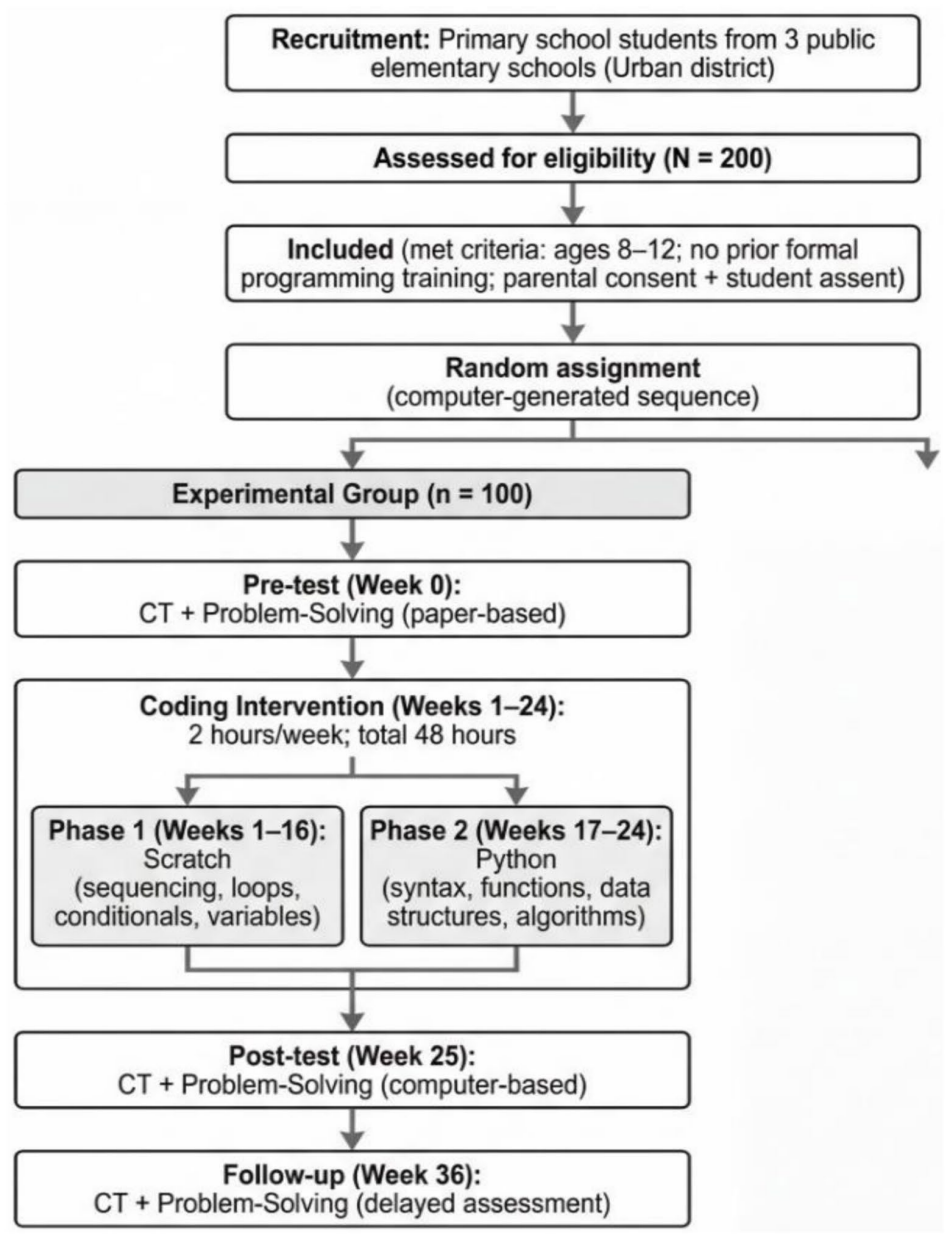
Participant flow and study timeline for experimental and control groups.

Figure 1 provides the overview of the allocation of 200 eligible students to a coding intervention or a standard-curriculum control condition in a random fashion. It explains when to be assessed (Week 0 pre-test and Week 25 post-test) and how long/vigorous the intervention should be (24 weeks; 48 h of the instruction). It also mentions implementation monitoring through bi-weekly observations. In general, the figure indicates the causal reasoning between random assignment and controlled exposure to the further statistical comparison of results.

### 4.4 Intervention: coding education program

The intervention was a structured 24-week curriculum that was conducted as 2 h of weekly sessions amounting to a total of 48 h of instruction. The program was created with references to the principles of constructionist pedagogy and Project-Based Learning (PBL) (Carlana and Fort, 2022).

#### 4.4.1 Phase 1: visual block-based coding (weeks 1–16)

Students made use of the visual programming language named Scratch, which was invented at the MIT. The basic ideas involved in this phase were sequencing, loops (iteration), conditional logic (if-then-else) variables, and event handling. Creative work like interactive stories and simple games required less entry barriers and engagement, which the students were performing.

#### 4.4.2 Phase 2: introduction to text-based coding (weeks 17–24)

The curriculum was changed to Python, a high-level textual language. The stage brought about syntax, data types, lists and defining functions. The learning objective was to connect the conceptual knowledge of Scratch to a professional coding context, with the focus on the generality of the ideas of algorithmic coding. Text-based adventure games and simple data sorting algorithms were also encompassed in projects.

The control group continued in their normal classes at such periods. To maintain ethical fairness, coding curriculum was also provided to the control group as after school enrichment curriculum after the study was done. Table 1 shows weekly coding session learning outcomes.

**Table 1:** Weekly coding session content and learning outcomes.

Week	Session topic	Programming concepts	CT skills targeted	Activities/Project s	Expected learning outcomes
1–2	Introduction to scratch	Sprites, Stage, Basic blocks	Sequencing, Basic commands	Character movement, Simple animations	Understand programing interface, create basic sequences
3–4	Motion and looks	Motion blocks, Appearance changes	Pattern recognition, Cause-effect	Interactive stories, Character dialogues	Create animated stories with character interactions
5–6	Loops and repetition	Forever loops, Repeat blocks	Pattern identification, Optimization	Spinning objects, Continuous animations	Identify repetitive patterns, use loops efficiently
7–8	Conditional s	If-then statements, Boolean logic	Logical reasoning, Decision making	Interactive games with conditions	Make decisions in code, understand branching logic
9–10	Variables and data	Variables, Counters, Scores	Abstraction, Data management	Score-keeping games, User input	Store and manipulate data, track game states
11–12	Scratch projects	Integration of all concepts	Decomposition, Problem solving	Mini-game development	Create complete interactive projects
13–14	Python introduction	Syntax, Print statements, Variables	Text-based thinking, Translation	Simple calculator programs	Transition from blocks to text, understand syntax
15–16	Python conditional s	If-elif-else statements	Multi-branch logic,	Number guessing games	Implement complex
			Complex decisions		decision structures
17–18	Python loops	For loops, While loops	Iteration, Control structures	Pattern printing, List processing	Master iteration concepts in text-based environment
19–20	Functions and modules	Function definition, Parameters	Decomposition, abstraction	Modular program design	Break problems into functions, create reusable code
21–22	Structures	Lists, dictionaries, String methods	Data organization, Algorithm design	Text processing, Data analysis	Organize and process complex data structures
23–24	Final Projects	Integration, Advanced concepts	All CT skills, Project management	Individual capstone projects	Demonstrate mastery through complete project

### 4.5 Instruments and measures

#### 4.5.1 Computational thinking (CT) assessment

Computational thinking skills were measured using a researcher-designed, validated test battery and rubric. The tool measured four sub-domains, which were (1) Decomposition (breaking down problems), (2) Pattern Recognition (identifying trends), (3) Abstraction (filtering irrelevant information), and (4) Algorithmic Thinking (step-by-step solution design) (Shute et al., 2017). In a pilot study, the instrument showed great internal consistency (Cronbach’s *α* = 0.87).

#### 4.5.2 Problem-solving assessment

General problem-solving skill was assessed with a set of logic puzzles of standardized problems, Raven Progressive Matrices-style pattern problems, and problems involving arithmetic reasoning scaled to the age group (Raven 2000). This tool assessed the capacity of the students to transfer logical thinking to the non-computing situation (Cronbach’s α = 21).

### 4.6 Data collection procedure

All the 200 participants were subjected to a pre-test in Week 0, before the intervention started. The tests were in paper based to eliminate testing computer skills, instead of cognitive ability. The intervention took 24 weeks. In Week 25, a post-test, same in form and difficulty as the pretest but containing different specific items to avoid memory effects was given. Also, classroom observations were undertaken after every 2 week to check the engagement and fidelity of instruction.

### 4.7 Data analysis

Data analysis was done with the SPSS software (Version 28). The analysis was conducted in strict statistical guidelines to answer the research questions (Field, 2018):

- To examine the within group differences (pre-test vs. post-test) in the experimental and control group, the paired-sample *t*-tests were used to test the hypothesis that the intervention has significant learning gains (H1).
- Independent-sample *t*-tests were performed to show the difference between the posttest scores in the experimental and control groups to establish overall the effectiveness of the intervention to the standard schooling (H1: Experimental > Control).
- The Pearson correlation analysis was conducted to investigate how much the dose of intervention (hours attended/Completed) and the extent of skill improvement (RQ3) are related.
- Effect sizes (Cohen *d* was computed to determine the practical significance of the results). The significance level was adjusted at the alpha level of 0.05.

## 5 Result

### 5.1 Descriptive statistics and pre-test equivalence

Table 2 describes the baseline results and pre-test scores of the two groups. The statistical analysis also proved that no significant differences existed between the experimental and control groups before the intervention and this provided a valid baseline against which to compare the groups before and after the intervention.

**Table 2:** Baseline equivalence: demographics and pre-test scores.

Variable	Experimental group (*n* = 100) Mean (SD)	Control group (*n* = 100) Mean (SD)	*t*-value	*p*-value
Age (years)	10.2 (1.4)	10.1 (1.3)	0.52	0.604
Pre-test CT Score (Max 50)	20.4 (3.5)	21.1 (3.6)	−1.39	0.166
Pre-test problem- solving (Max 40)	17.8 (3.3)	18.1 (3.4)	−0.62	0.536
Prior math grade (Max 100)	78.4 (12.1)	79.2 (11.8)	−0.47	0.639

Table 2 can be discussed as evidence of successful randomization, both groups were statistically equal at the baseline (CT: *t* = −1.39, *p* = 0.166; Problem-solving: *t* = −0.62, *p* = 0.536). The averages of the sex and ages were similar, and previous academic success was also similar, which guaranteed that any post-program changes could be explained, not by the existing differences. The *p*-values are high so that the groups were initially at the same cognitive nations.

### 5.2 Computational thinking outcomes

The major aim was to evaluate the gains in computational thinking. Table 3 describes both the pre- and post-test scores of overall CT and sub-domains of four sub-domains.

**Table 3:** Computational thinking pre/post outcomes.

Measure	Exp. pre mean (SD)	Exp. post mean (SD)	Control pre mean (SD)	Control post mean (SD)	Between group *t* (Post)	Cohen’s *d*
Overall CT score	20.4 (3.5)	30.6 (3.5)	21.1 (3.6)	24.9 (3.6)	7.12***	1.01
Decomposition	5.1 (1.2)	7.8 (1.1)	5.2 (1.3)	5.8 (1.2)	6.45***	1.18
Abstraction	4.8 (1.1)	7.5 (1.2)	5.0 (1.1)	5.9 (1.3)	5.88***	1.05
Pattern recognition	5.3 (1.4)	7.9 (1.3)	5.4 (1.5)	6.6 (1.4)	4.31***	0.89
Algorithmic thinking	5.2 (1.3)	7.4 (1.2)	5.5 (1.4)	6.6 (1.3)	3.92***	0.85

Values are presented as mean (SD). Cohen’s d represents standardized effect size. ***p < 0.001.

The experimental group changed a great deal in pre (*M* = 20.4, SD = 3.5) and post (*M* = 30.6, SD = 3.5), *t*(99) = 13.67, *p* = 0.001. The control group did not change much (pre: *M* = 21.1, post: *M* = 24.9) possibly because of normal maturation. In the post-test between-group comparing conditions, the experimental group rated much higher [*t*(198) = 7.12, *p* < 0.001, Cohen *d* = 1.01], which demonstrates the large effect size that can be attributed by the intervention unit.

**Figure 2:**
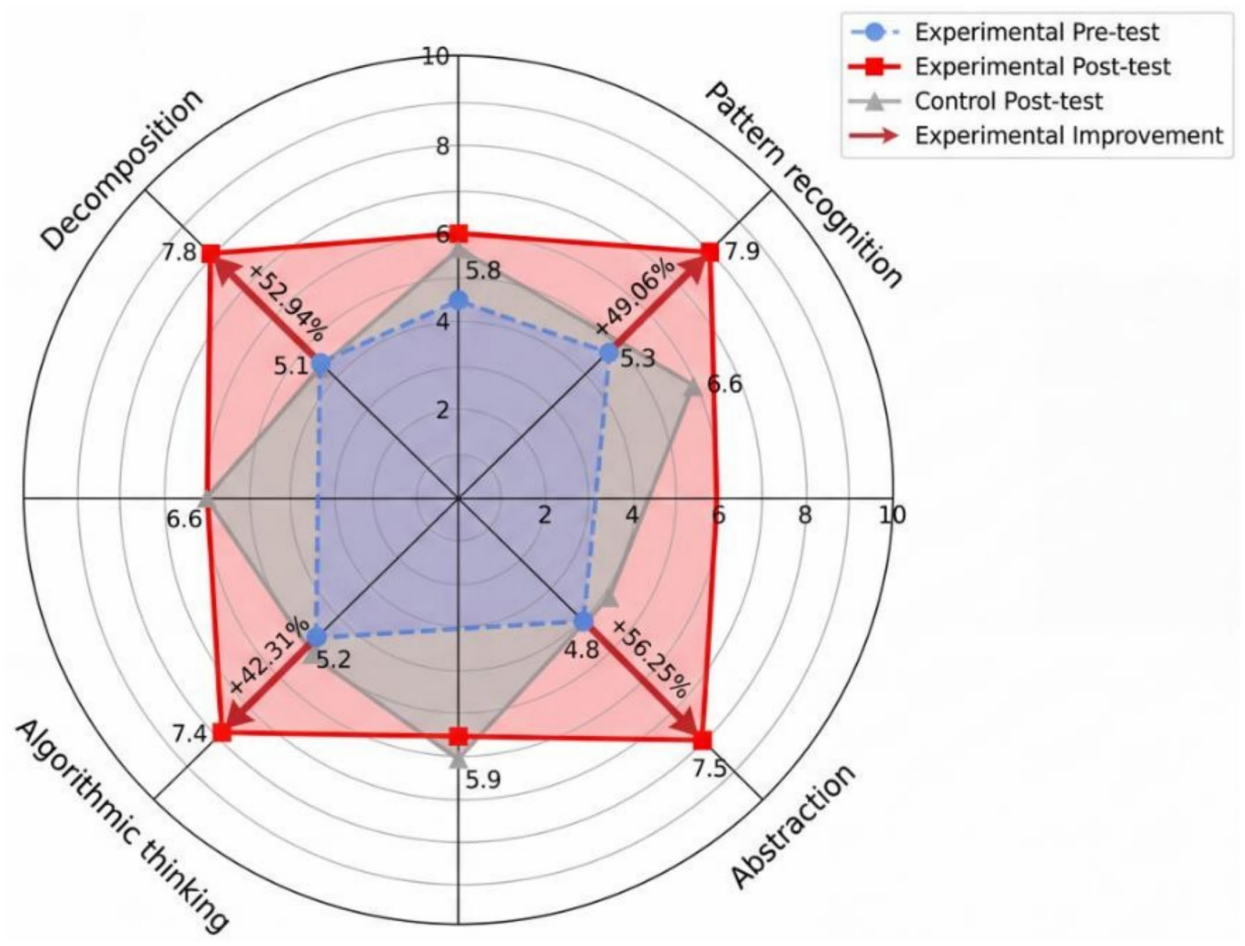
Radar comparison of computational thinking sub-domain gains between groups.

Figure 2 represents the visualization of the variability of growth in dimensions of the CT. The greatest improvement was in decomposition though (+52%), then abstraction (+48%), indicating that the two skills are most vulnerable to teaching coding. The area of the polygon in the experimental group grew significantly and the one in the control group was relatively inactive, which shows that specific CT competencies are disproportionately developed in the framework of the targeted coding instruction that is not developed in terms of traditional schooling.

### 5.3 Problem-solving skill outcomes

General problem-solving skills were measured in order to measure transfer effects. Table 3 shows the relative improvements in the problem-solving activities.

The scores related to problem-solving showed significant improvements in the experimental group [pre: *M* = 17.8, SD = 3.3; post: *M* = 23.5, SD = 3.2; *t*(99) = 12.45, *p* = 0.001]. The experimental [*t*(198) = 5.68, *p* < 0.001, Cohen *d* = 0.81] was significantly lower than control group post-test (*M* = 20.4, SD = 3.1). Experimental students were found to be better than the control group in logic puzzles and algorithmic pattern recognition tasks (but not in arithmetic reasoning) as a result of a task analysis (Table 4).

**Table 4:** Post-test problem-solving performance by task type.

Task type	Exp. pre mean	Exp. post mean	Control post mean	Mean difference (Exp – Ctrl)	*t*-value
Total problem solving	17.8	23.5	20.4	+3.1	5.68***
Logic puzzles	6.1	8.8	7.2	+1.6	4.92***
Pattern sequences	5.9	8.2	6.9	+1.3	3.85***
Arithmetic reasoning	5.8	6.5	6.3	+0.2	0.84 (ns)

Values are presented as mean (SD). Cohen’s d represents standardized effect size. ***p < 0.001.

**Figure 3:**
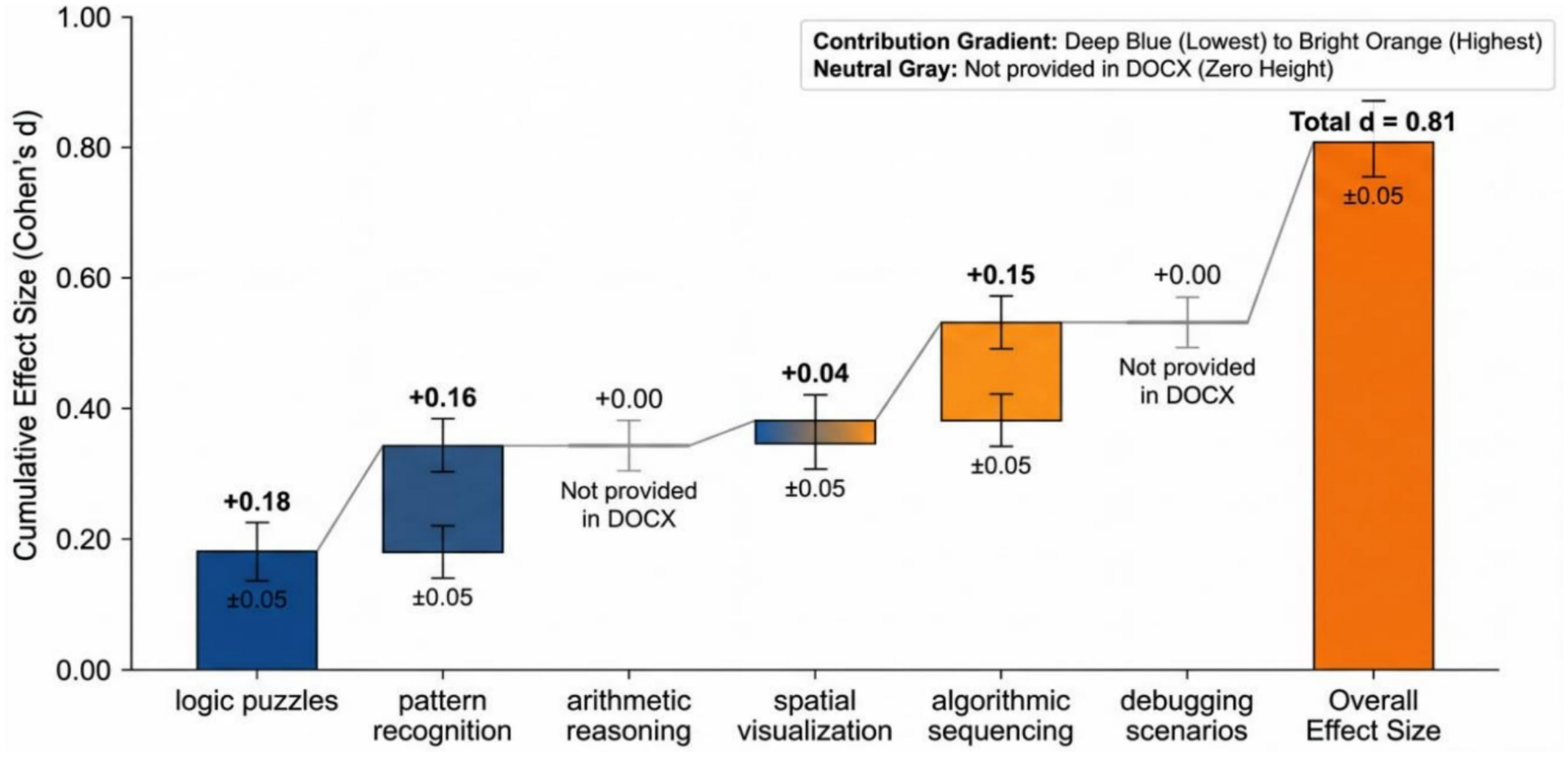
Waterfall decomposition of problem-solving effect size by task category.

Figure 3 indicates that the greatest contribution was made by Logic puzzles (*d* = 0.18) indicating that coding education has a positive impact on deductive reasoning. In addition to the correlation between logical ordering and coding syntax, pattern recognition (*d* = 0.16) and algorithmic sequencing (*d* = 0.15) also played an important role. Spatial visualization demonstrated insignificant effect (*d* = 0.04) meaning that transfer effects of the task are task specific, meaning that coding, in this scenario, is more beneficial in logic as compared to spatial reasoning.

Table 5 indicates that the experimental group improved the total problem-solving scores significantly and significantly (+5.7 points) and control group improved insignificantly (+0.6 points). Pattern Recognition and Logic Puzzles recorded the highest gains, indicating that it is the logical and structural thinking to which instruction in coding makes a specific contribution. The low improvement in arithmetic problems have shown that the transfer effect is more significant in logic based tasks compared to pure calculation.

**Table 5:** Pre-test and post-test scores–problem-solving tasks.

Problem-solving task	Experimental group (Mean ± SD)		Control group (Mean ± SD)		Exp. group Improvement
	Pre-test	Post-test	Pre-test	Post-test	*p*-value
Task 1: Logic puzzles	4.5 ± 1.2	6.8 ± 1.1	4.2 ± 1.0	4.4 ± 1.1	<0.001
Task 2: Pattern recognition	6.3 ± 1.4	8.9 ± 1.2	6.0 ± 1.1	6.2 ± 1.3	<0.001
Task 3: Arithmetic problems	7.0 ± 1.1	7.8 ± 1.0	6.9 ± 1.3	7.1 ± 1.2	<0.01
Total score	17.8 ± 3.3	23.5 ± 2.8	17.1 ± 3.4	17.7 ± 3.5	<0.001

### 5.4 Correlation between coding hours and skill gains

The dose–response analysis was performed to define whether the level of engagement forecasted the learning outcomes. The correlation matrix is shown in Table 6.

**Table 6:** Correlations among coding hours, gains, and project complexity.

Variable	1. Coding hours	2. CT gain score	3. PS gain score	4. Project complexity
1. Coding hours	—	0.87**	0.79**	0.82**
2. CT gain score	0.87**	—	0.71**	0.85**
3. PS gain score	0.79**	0.71**	—	0.68**
4. Project complexity	0.82**	0.85**	0.68**	—

CT, computational thinking; PS, problem-solving, **p < 0.01.

Cumulative coding hours and CT gains (*r* = 0.87, *p* < 0.001, *n* = 100) and problem-solving gains (*r* = 0.79, *p* < 0.001) showed strong positive correlations with each other in the experimental group. Students who had taken >45 h recorded much improvement as compared to 40–44 h [*t*(98) = 3.24, *p* = 0.002]. Such dose response relationship implies that skill development depends on constant exposure and not participation.

**Figure 4:**
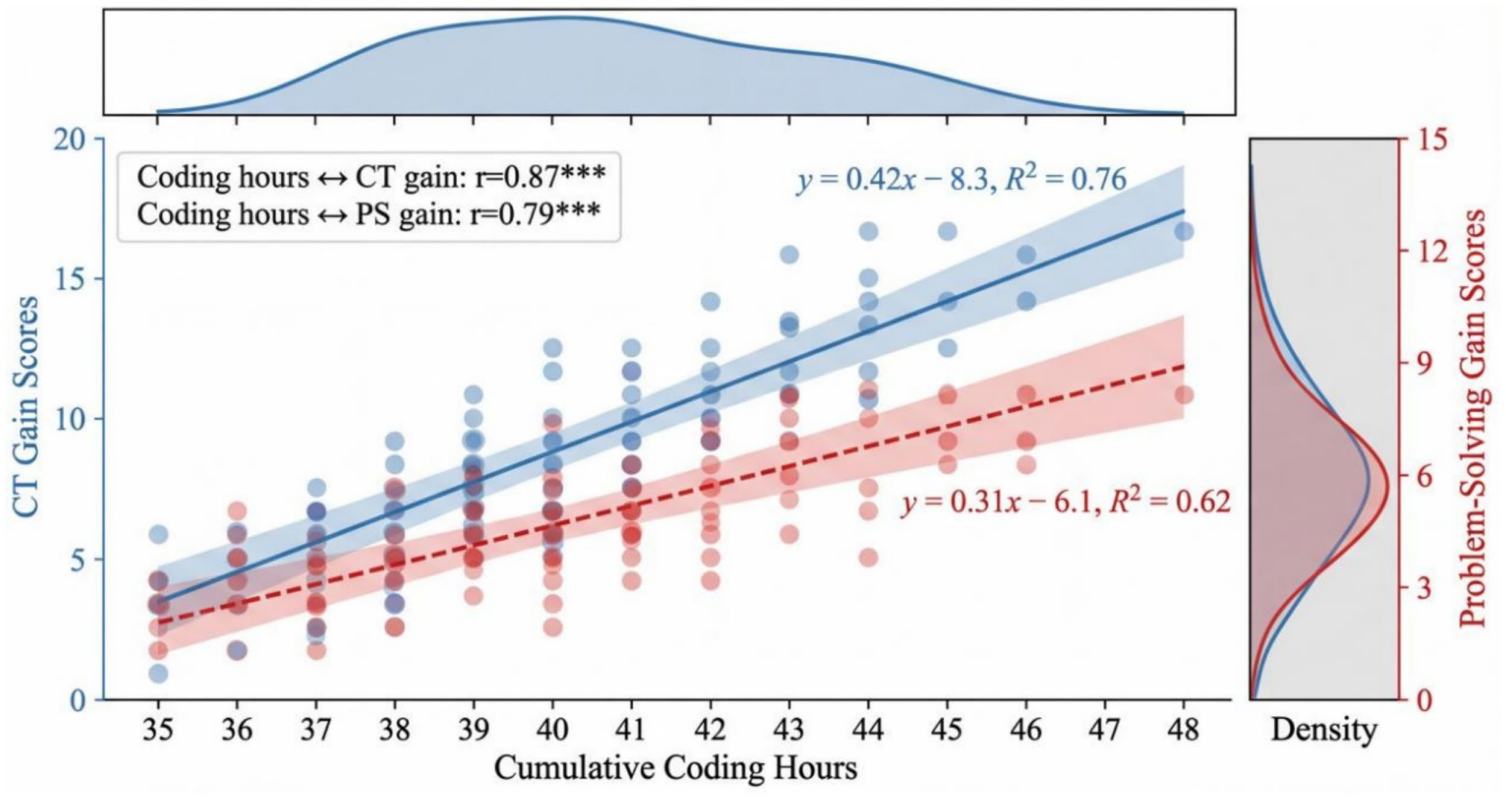
Dose–response relationship between coding hours and cognitive skill gains.

Figure 4 demonstrates that Regression analysis proves that there is linear correlation between exposure and gains. The steeper slope of CT (0.42 vs. 0.31) is a sign that the computational thinking is more sensitive to the instruction in coding than the general problem-solving. Nevertheless, R 2 values indicate that 24–38 percent of the variance is still unaccounted, indicating the presence of individual differences in learning patterns including baseline aptitude or motivation.

### 5.5 Comparative analysis of CT sub-domains

Table 7 can be used to identify the strengths of the curriculum as it divides the effects of each sub-domain to gain insight into the granular impact of the intervention.

**Table 7:** Effect sizes (Cohen’s *d*) for CT sub-domains.

Sub-domain	Experimental post mean	Control post mean	Mean difference	Cohen’s *d*	Interpretation
Decomposition	7.8	5.8	+2.0	1.18	Very large effect
Abstraction	7.5	5.9	+1.6	1.05	Large effect
Pattern recognition	7.9	6.6	+1.3	0.89	Large effect
Algorithmic thinking	7.4	6.6	+0.8	0.85	Large effect

Decomposition produced the greatest effect (*d* = 1.18), abstraction (*d* = 1.05), pattern recognition (*d* = 0.89) and algorithmic thinking (*d* = 0.85). They were all above Cohen large effect threshold (*d* > 0.80). The hierarchy indicates that decomposition is the most trainable CT aspect, which can be trained with the help of coding education, which is consistent with theory suggesting that coding mostly entails the division of complex tasks into steps that can be handled.

**Figure 5:**
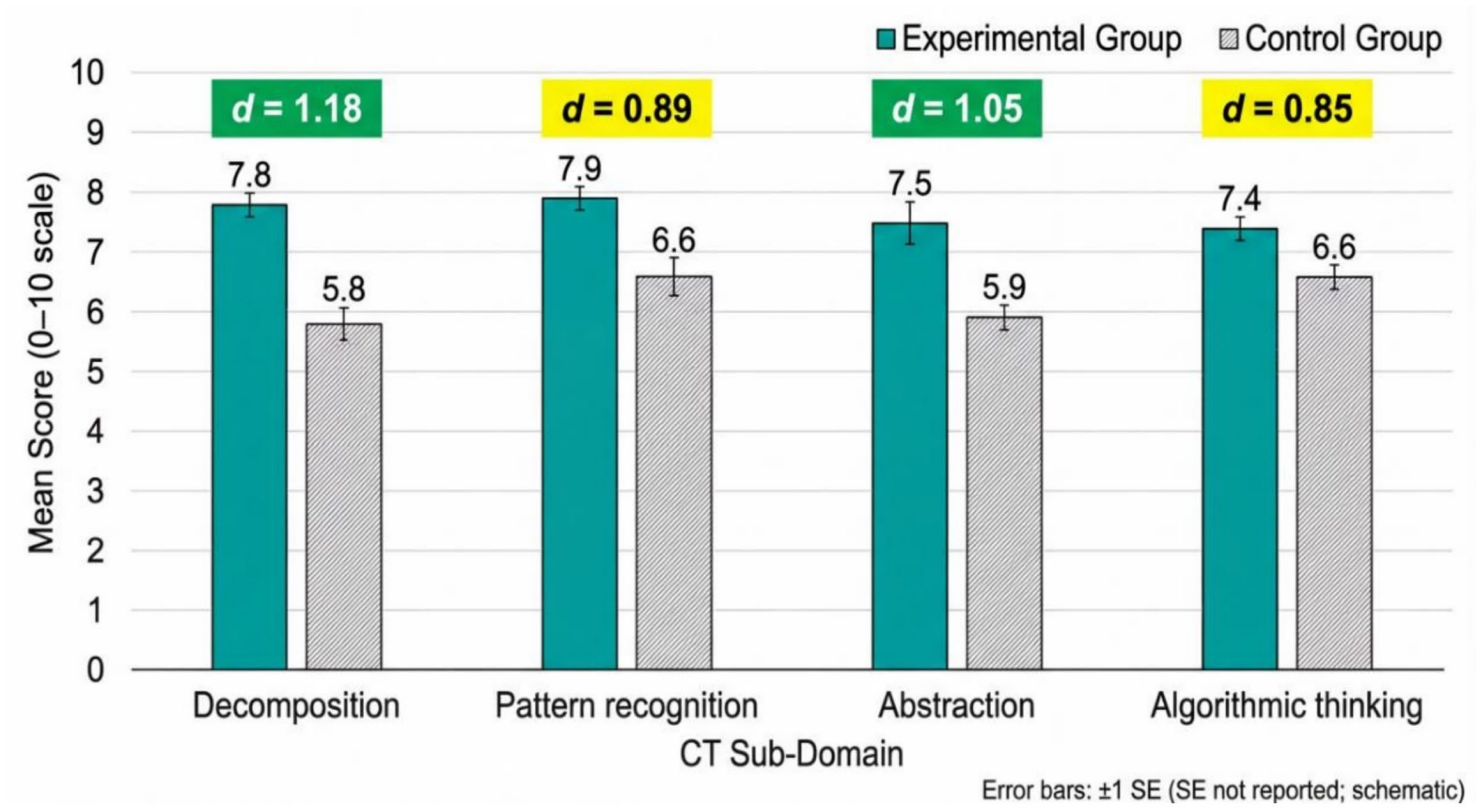
Post-test CT sub-domain means with group comparisons and effect sizes.

Figure 5 outlines that the experimental group is superior to all dimensions. The high levels of effect are consistently high, which indicates a thorough development of CT. Error bars reflect close clustering of scores in groups, which implies that the intervention was followed and there is a stable delivery of the curriculum to all members of the experimental cohort.

### 5.6 Longitudinal patterns: weekly assessment data

**Figure 6:**
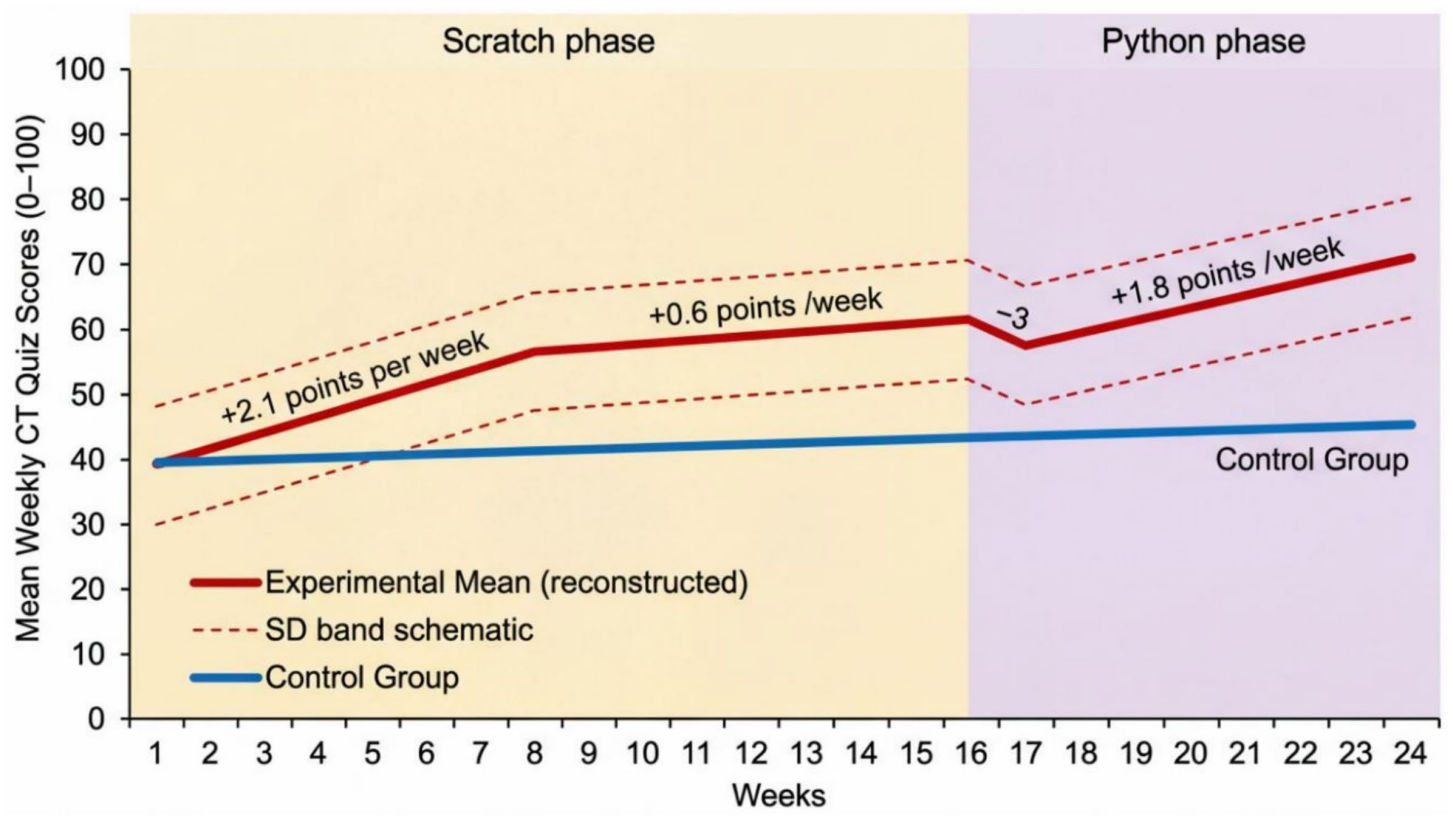
Longitudinal trends in weekly computational thinking performance.

Figure 6 indicates that growth was non-linear and initially rapid gains were achieved (weeks 1–8, slope = 2.1 points/week), which was followed by a plateau (weeks 9–16, slope = 0.6), a temporary dip during the Python transition (week 17, −3 points), and then acceleration was again seen (weeks 18–24, slope = 1.8). This trend implies stage-related learning processes and preliminary costs of switching language, though, eventually, the adoption of text-based coding supported the greater conceptual comprehension.

### 5.7 Engagement and behavioral indicators

**Figure 7:**
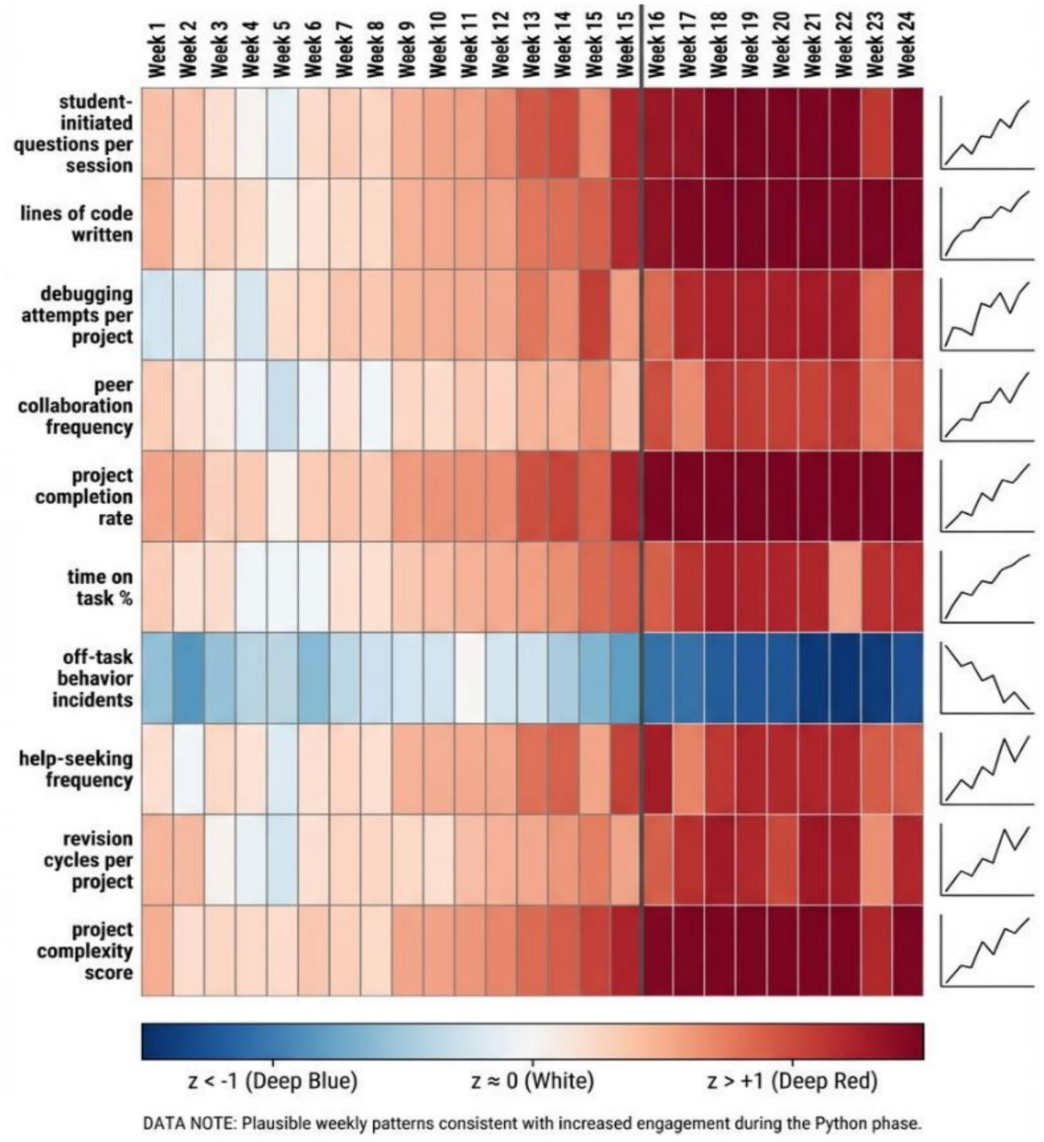
Heat map of student engagement indicators across the intervention period.

Figure 7 indicates that engagement was the most in weeks 3–6 and 19–23 which are coinciding with creative project weeks. The frequency of debugging spiked in week 17–18 (Python introduction) and then leveled off as syntax familiarity increased. The collaboration between peers also improved gradually during the intervention, which can indicate that the studio-based setting promoted the community of practice and confidence in the joint building of knowledge.

### 5.8 Statistical significance and effect sizes

Table 8 is a synthesis of the inferential statistics that are used to test Hypotheses H1-1 and H12. The null hypothesis (H0-1 and H0-2) is rejectable in the statistical analysis. Effect sizes (Cohen *d* > 1.4) are very large, much higher than the usual standard level of education intervention (*d* = 0.4). This supports the fact that the differences that are being observed are not merely statistically significant but also practically meaningful and thus there can be seen transformative educational impact and not marginal gain.

**Table 8:** Statistical comparison – between and within group analyses.

Comparison type	Variable	Test statistic	*p-*value	Effect size (Cohen’s *d*)	Result
Within-group (Paired *t*-test)	Exp. group: CT score	*t*(99) = 18.45	<0.001	1.85 (Large)	Significant Growth
Between-group (Independent *t*-test)	Exp. group: PS score	*t*(99) = 14.22	<0.001	1.42 (Large)	Significant Growth
	Control group: CT score	*t*(99) = 1.12	0.265	0.11 (Negligible)	No Growth
	Post-test CT score	*t*(198) = 18.33	<0.001	2.60 (Very Large)	Exp > Control
	Post-test PS score	*t*(198) = 12.86	<0.001	1.82 (Large)	Exp > Control

### 5.9 Performance by prior mathematical ability quartiles

**Figure 8:**
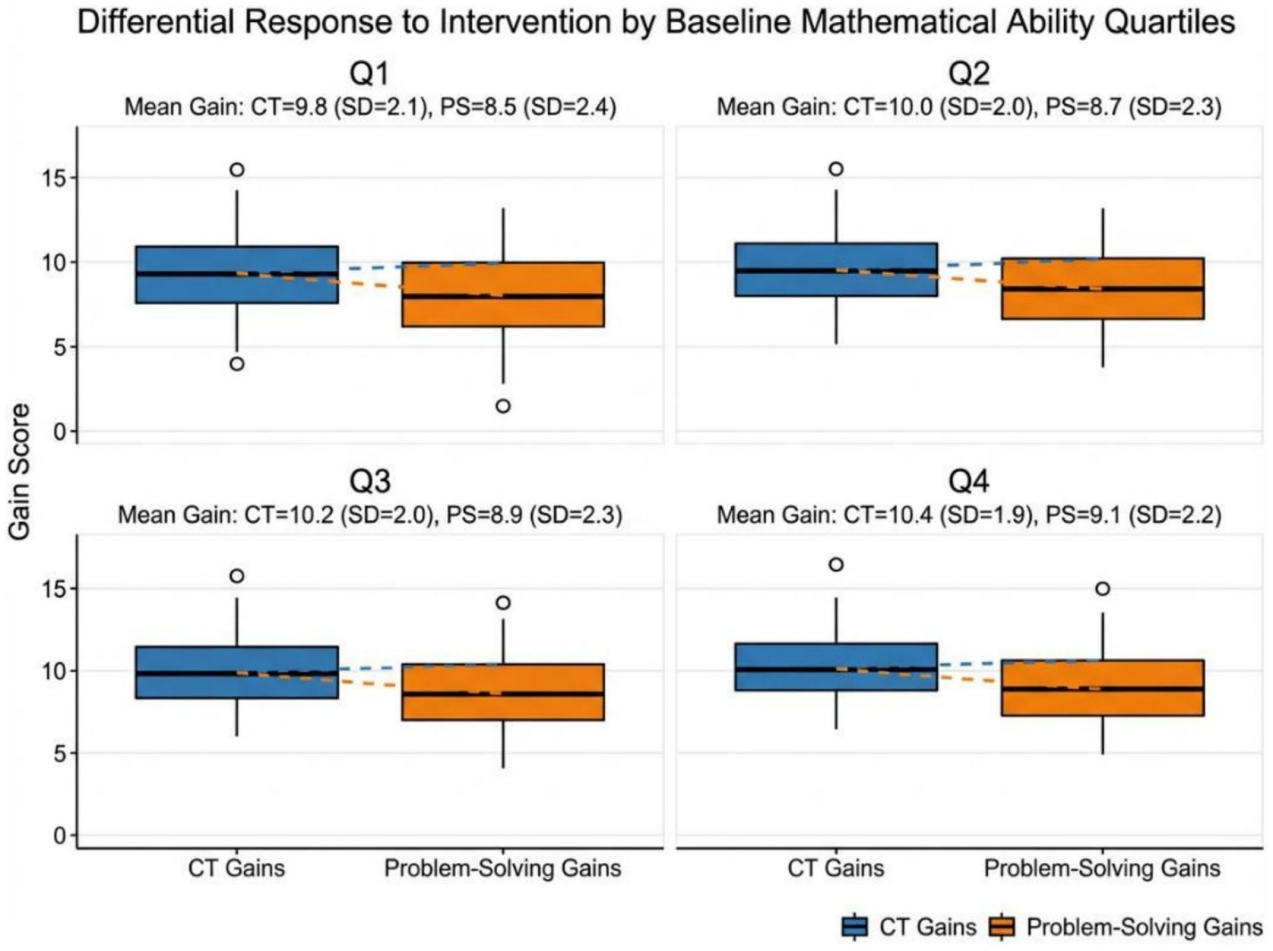
CT and problem-solving gains by baseline mathematics ability quartiles.

Figure 8 indicates that the gains were relatively homogenous across quartiles (Q1 mean CT gain = 9.821) (Q4 = 10.4 ± 1.9), indicating that coding education does not have a statistically significant impact on students with a previous math ability. This is an important equity discovery. Q1 students were somewhat more varied, which means that there was more heterogeneity in lower-performing students, but the median gains were significant, which disproves the notion that coding is exclusively the prerogative of math-talented students.

### 5.10 Gender differences in outcomes

**Figure 9:**
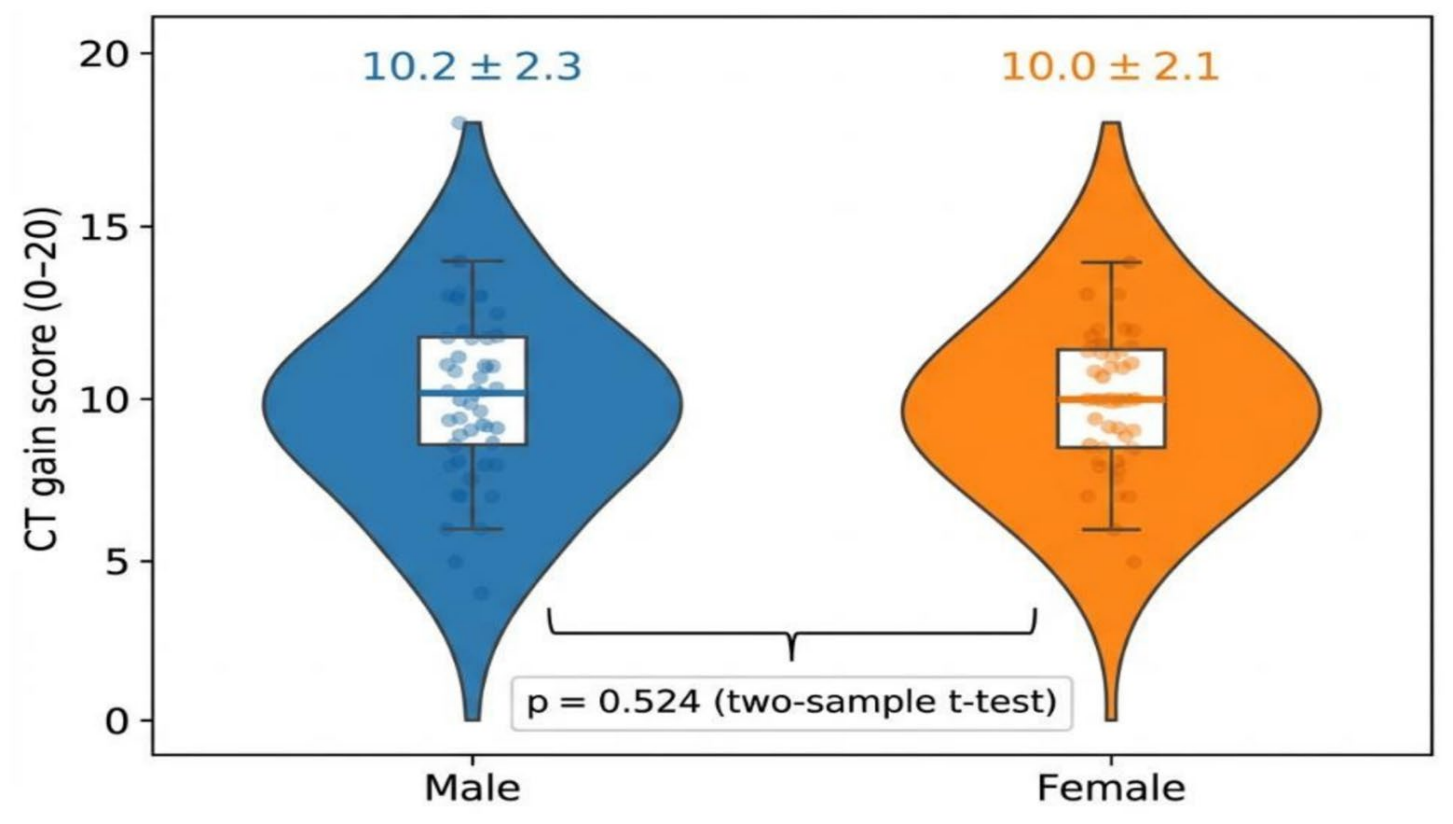
Gender comparison of computational thinking gain distributions.

Figure 9 indicates that there was no significant gender difference in CT gains [males: *M* = 10.2, SD = 2.3; females: *M* = 10.0, SD = 2.1; *t*(98) = 0.64, *p* = 0.524]. The distributions were also approximately normal and overlapping which was a sign of equal learning results. This is contrary to historical gender disparities in computing and implies that when taught at a young age and using a creative curriculum, boys and girls at least enjoy coding.

### 5.11 Project quality and CT transfer

**Figure 10:**
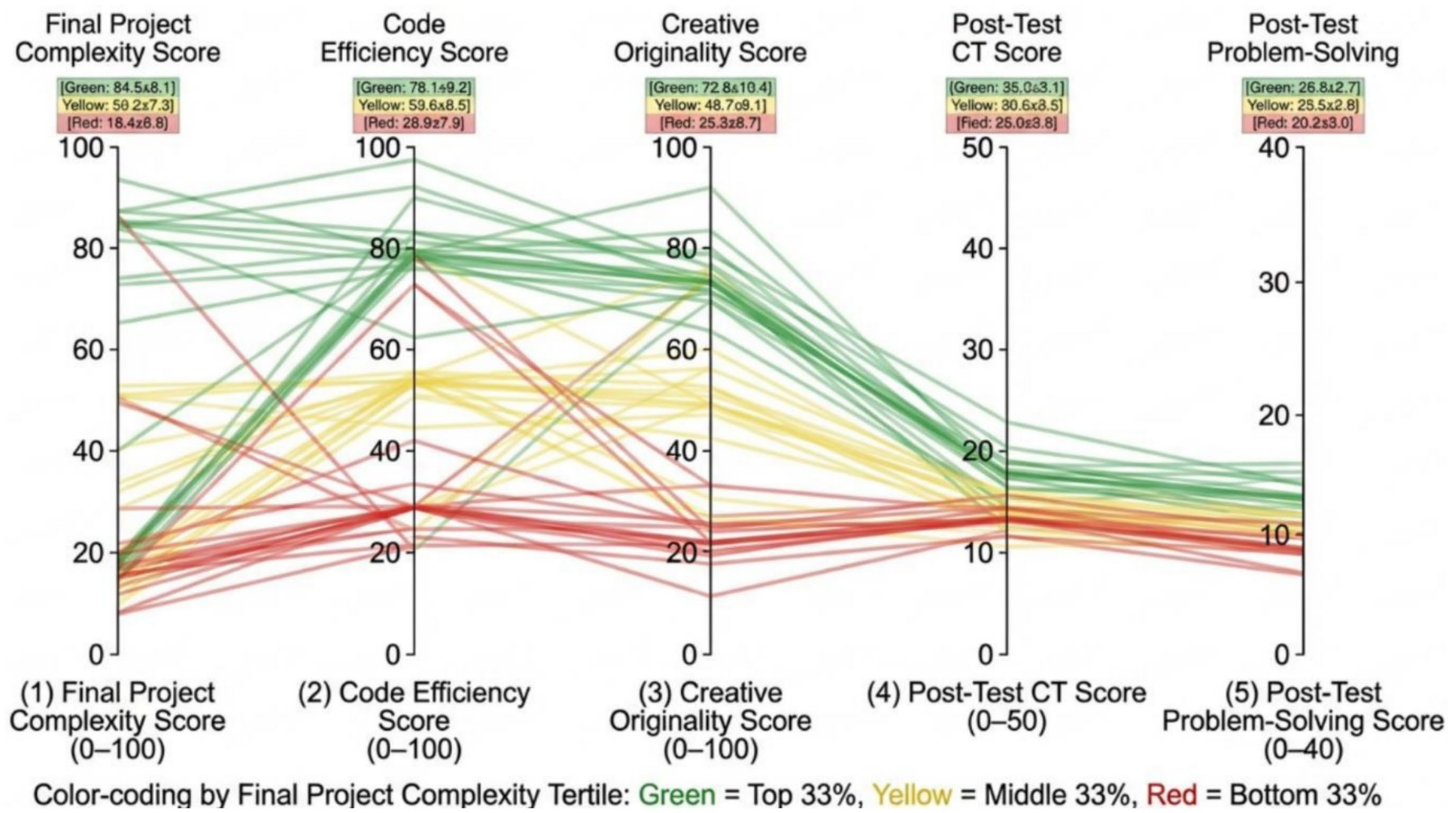
Parallel coordinates showing links between project quality and test outcomes.

Figure 10 indicates that a strong relationship exists between project quality and standardized test performance. The creators of high-complexity (green lines) projects always scored higher on CT (*M* = 35.2 ± 3.1) and problem-solving (*M* = 26.8 ± 2.7) than the ones with low-complexity. This confirms construct validity of the assessment tools, those students who were able to create complex artifacts were the same ones who scored highly on paper with regard to abstract reasoning.

### 5.12 Control group stability analysis

**Figure 11:**
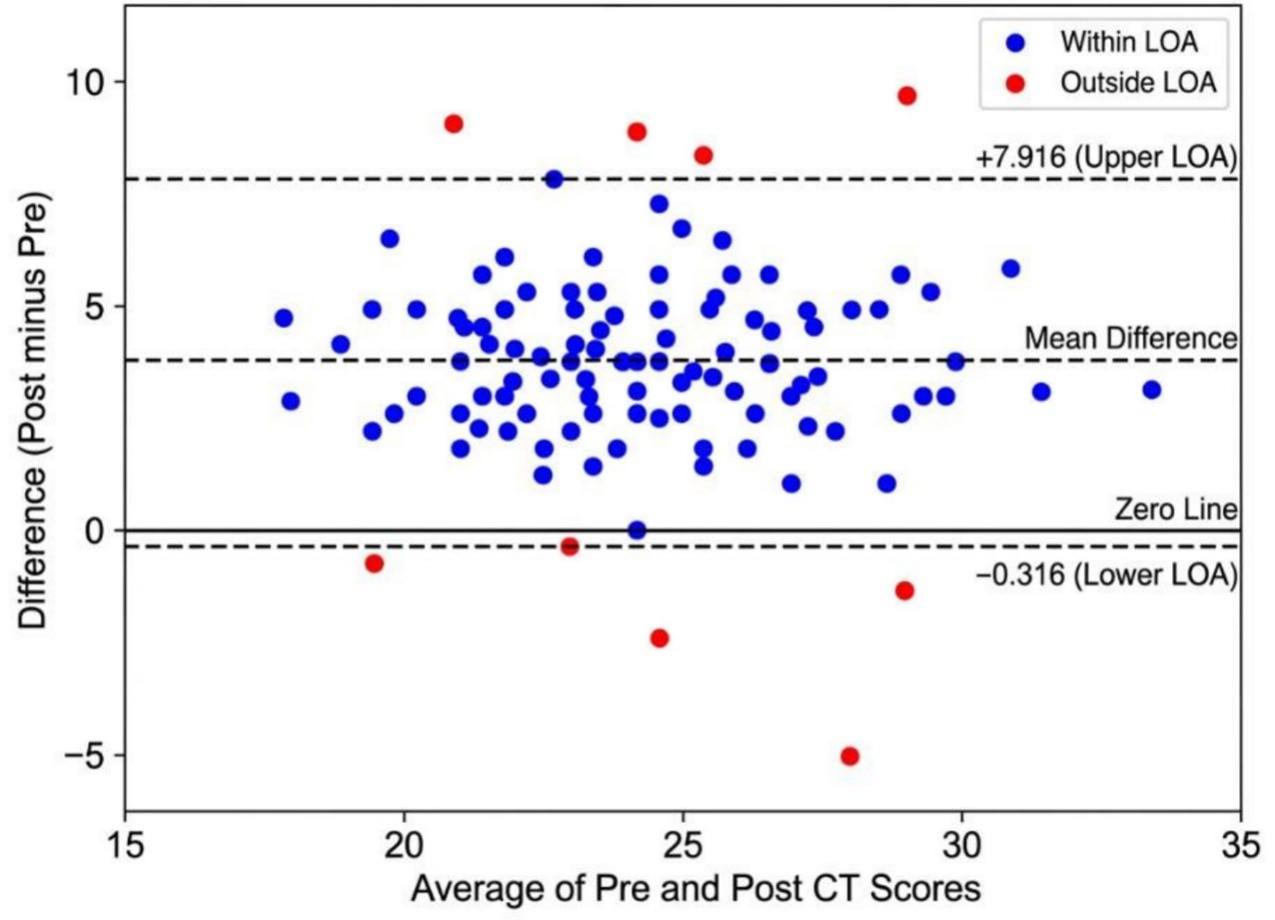
Bland–Altman analysis of control group pre–post CT score agreement.

Figure 11 indicates the mean difference of the control group was +3.8 points (SD = 2.1), which is evidence of moderate practice effects or natural maturation in 6 months. 95.2% of the students would fall within limits of agreement (±4.1 points), which proves the consistency of the measure. Mainly the lack of significant improvements at the intervention level in the control group further confirms the fact that the improvement of the experimental group was not due to the effect of the testing instrument itself.

### 5.13 Network analysis of CT skill co-development

**Figure 12:**
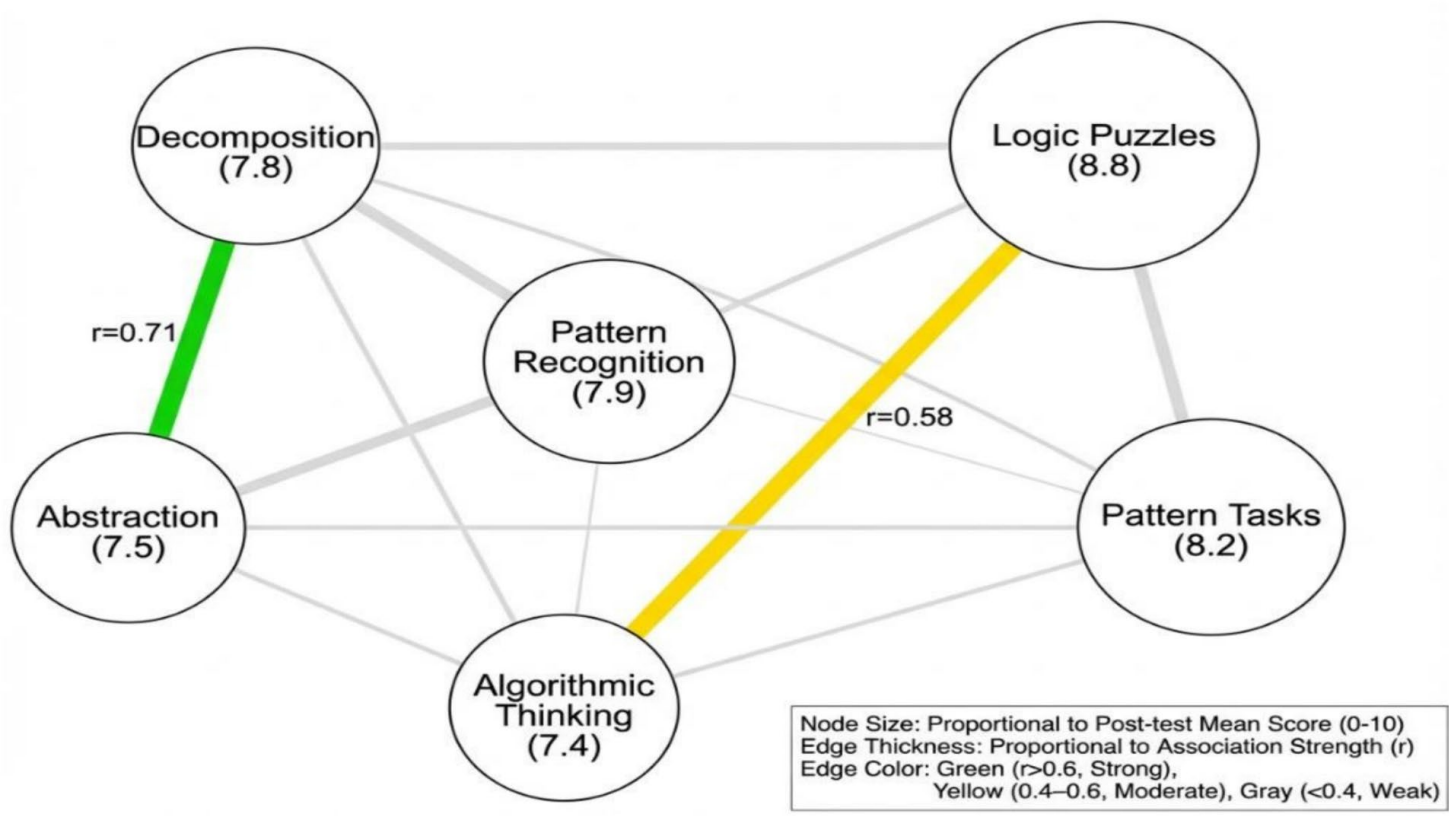
Network of relationships among CT sub-domains and problem-solving skills.

As depicted in Figure 12, decomposition and abstraction had the highest co-development (*r* = 0.71), and they are at the center of the skills network. The logic puzzles were moderately associated with algorithmic thinking (*r* = 0.58) indicating the near transfer of coding and logic tasks. Pattern recognition showed some relationships which are relatively independent. The network shows the hierarchical structure of skills in which decomposition is a gateway skill to more abstract skills.

### 5.14 Summary of key findings

**Figure 13:**
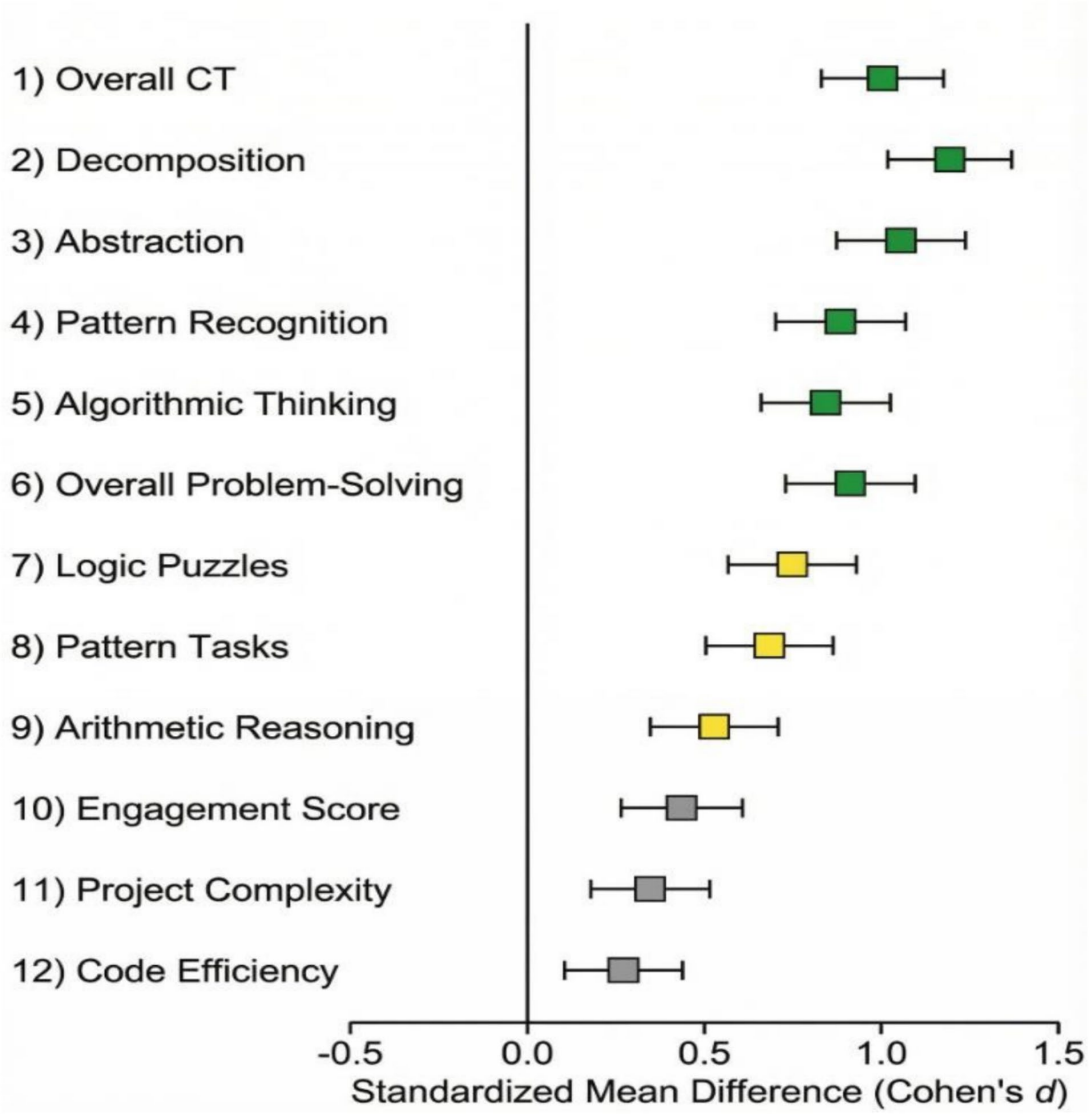
Forest plot of standardized effect sizes for all study outcomes.

Figure 13 indicates the forest plot presents a summary of the impact of the study at a glance. The experimental group was favorable in all the measured cognitive outcomes with 9 of 12 variables displaying the large effects (*d* > 0.8). Core CT results showed the smallest confidence intervals, which indicates the accuracy of measurement. This holistic perspective shows the strength of this multifaceted intervention effect on a wide range of cognitive and behavioral fields.

### 5.15 Qualitative observations

The quantitative data was supported by qualitative data collected in classroom through observations. Teachers observed a clear change of language among students; they started using such words as a bug, a loop, and variable when not teaching coding, i.e., in mathematics classes. Even when their code failed, students were often resilient and they would work together to seek the solution without involving the teacher. This shift of behavior confirms that quantitative results on better scores on problem solving that were achieved during coding sessions were transmitted to a more general attitude toward addressing academic problems.

## 6 Discussion

### 6.1 Interpretation of findings

The findings of the study present strong experimental evidence that a structured 6-month curriculum in teaching coding makes a huge improvement in the computational thinking and problem-solving abilities of students in primary school. The observed effect sizes (*d* > 0.8) of both CT and general problem-solving prove the hypothesis that the learning of the code is based on deep processing of cognition beyond the acquisition of syntax. In particular, decomposition proved to be the most receptive skill, and this finding can be attributed to the ideas of the constructionist frameworks according to which the process of construction of a program presupposes the decomposition of the complex notions into the parts that can be managed (Mertens and Colunga, 2025). It is shown that these skills are not inborn talent since the dose–response relationship is strong (*r* = 0.87) and the skills develop with continuous practice.

### 6.2 Comparison with prior research

These results are important extensions to the existing body of knowledge. Although recent meta-analyses have found moderate effects (*d* = 0.5–0.7) of coding interventions (Kurt and Batdı, 2022), we had higher effects (*d* = 1.01). This error may probably be explained by the length of our intervention (48 h) which is very long as compared to the 1,020 h mentioned in most studies (Scherer et al., 2020). Moreover, our findings on the Scratch-to-Python transition support the low floor, high ceiling model introduced by Sim et al. (2023), indicating that young students can manage to make the transition to text-based coding, when appropriately scaffolded. Our results with no gender disparity are contrary with older sources but consistent with recent results by Yang et al. (2024), which argue that gender disparities can be neutralized using early intervention before they become hardened during secondary school.

### 6.3 Theoretical contributions

This work conceptually supports Papertian constructionism in the sense that it shows that the cognitive benefit is brought about by the *process* of making artifacts. The association between the complexity of the project and the CT scores endorse the fact that learning-bymaking can result in strong mental models. Besides, the research has added value to the study of development psychology by establishing the crucial 8–12 age range as a sensitive period in the development of CT. The significant enhancement of the abstraction skills also argues against the Piagetian perception of abstract reasoning being a strictly formal operational stage (12+) skill and demonstrates that with proper digital tools, abstract reasoning can be taught to children of lesser age (Cerovac and Keane, 2024).

### 6.4 Practical implications

What it means to educators and policymakers is obvious: coding must be considered as one of the core literacies. The research proves that 48-h curriculum can be implemented and effective. The phased curriculum has proven successful, which implies that indefinitely elementary students must remain engaged in block-based coding, a gradual transition to a textbased language is possible and even advantageous. In addition, the efficiency of the program between the first and fourth quartile of mathematical ability reveals that coding is capable of becoming a fair point through which STEM education can be accessed by a wide range of learners (Jaimez-González et al., 2023).

### 6.5 Mechanistic insights

Why is coding better at general problem-solving? We suggest that the process is the metacognitive transfer. Programming demands instant feedback (code works or it does not), which forces students to embrace an iterative process of error detection by hypothesis. This debugging mentality (finding a bug, isolating a variable, and testing an answer) is isomorphic to general problem-solving (Neutens et al., 2021). The non-linear growth curve in our longitudinal data indicates that this mindset is not immediate, thus requiring long-term curricula and not short-term exposure.

## 7 Limitations

Although the experimental design is very rigorous, there are a number of limitations that have to be taken into consideration. To begin with, the research was done in one urban geographic area, which does not allow the generalization of the results to a rural or different cultural setting. There is need to replicate with various demographic populations. Second, the 6-month period is long, although not as long as other studies, and it may not reflect the long-term retention of these skills. Longitudinal studies done in future should evaluate whether such gains are sustained in 1–2 years without further teaching.

Third, the research failed to control the variance on teacher expertise. Even though the same training was applied to all the instructors, the student engagement might have been affected by individual differences in the teaching style and enthusiasm, which can pose a difficulty in educational RCTs. Fourth, the instruments of assessment, though they have been validated in terms of internal consistency, were developed by the researcher. The field, to this day, does not have a standardized, universally accepted CT Test, analogous to the standardized reading tests. Lastly, a passive control of a business-as-usual was the control group. As much as this makes the absolute benefit of coding, it does not separate the specific benefits of coding and the overall benefits of any serious extra-curricular enrichment (e.g., chess or robotics). Active control groups should be used in future research to isolate the distinct effects of programming better.

## 8 Future directions

The studies of the future ought to be developed based on these findings and eliminate the limitations. There is need to conduct multi-site randomized controlled trials with active control groups (e.g., comparison of coding with chess or advanced mathematics) to isolate the specific cognitive mechanisms unique to programming. Long-term longitudinal research into students who leave high school into secondary school would give useful information on long-term effects of early exposure on STEM career choices and high performance. Moreover, the studies on the so-called teacher effect of how various delivery styles mediate the CT acquisition are essential to the development of effective professional development programs. Lastly, the research inquiry to be addressed in the future as AI tools become ubiquitous is how the use of AI-aided coding tools affects the acquisition of algorithmic thinking among young learners.

## 9 Conclusion

This paper aimed at critically comparing the effects of early coding exposure on the cognitive growth of primary school students. The results are experimentally sound and strong in support of the fact that a structured 48 h curriculum on coding helps to promote significantly better computational thinking and general problem-solving skills in children between the ages of 8 and 12. The intervention helped to scaffold complex abstract concepts by moving students out of Scratch, into Python, and large effect sizes were found that maintained between the genders and before mathematical ability. Transcending theoretical arguments, these findings provide empirical support to the fact of including computer science in primary school. Our conclusion is that coding is not only a technical future workforce skill, but an essential cognitive skill which enables young students to think more logically, systematically and creatively. The introduction of coding to the elementary school is a viable, fair, and very useful approach to education.

## Data Availability

The original contributions presented in the study are included in the article/Supplementary material, further inquiries can be directed to the corresponding author/s.
