# Supplementary material for: Enhancing computational thinking through coding education in primary school students: an experimental study on the impact of early programming exposure on problem-solving skills
Source: Front Psychol. 2026 Feb 18;17:1734482. doi: 10.3389/fpsyg.2026.1734482 (PMC12990067; doi:10.3389/fpsyg.2026.1734482)

**Appendix**

**Appendix A: Student Feedback Questionnaire**


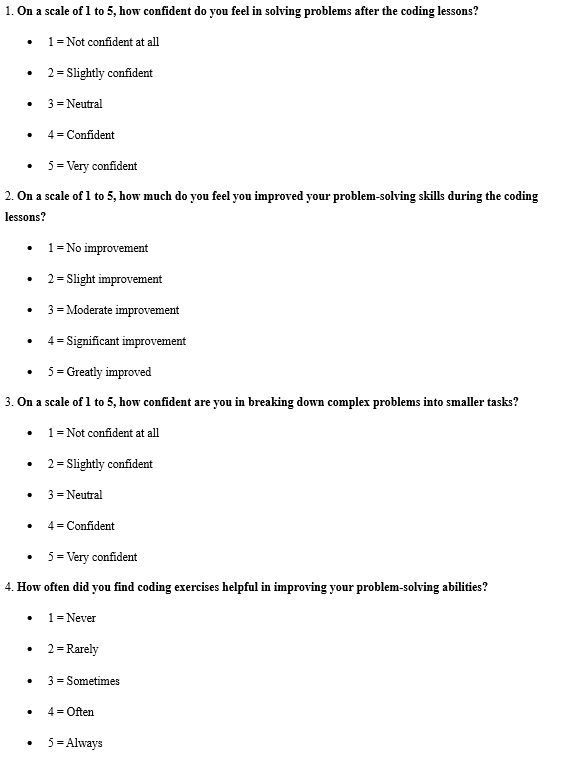


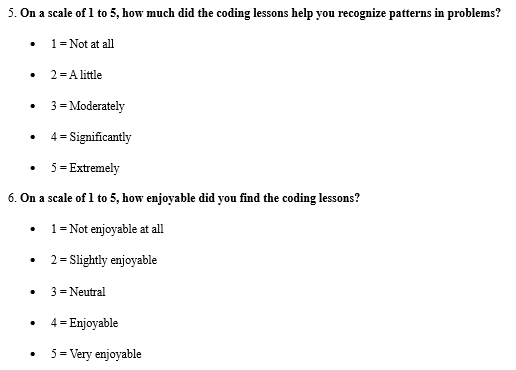


**Appendix B: Teacher Feedback Questionnaire**


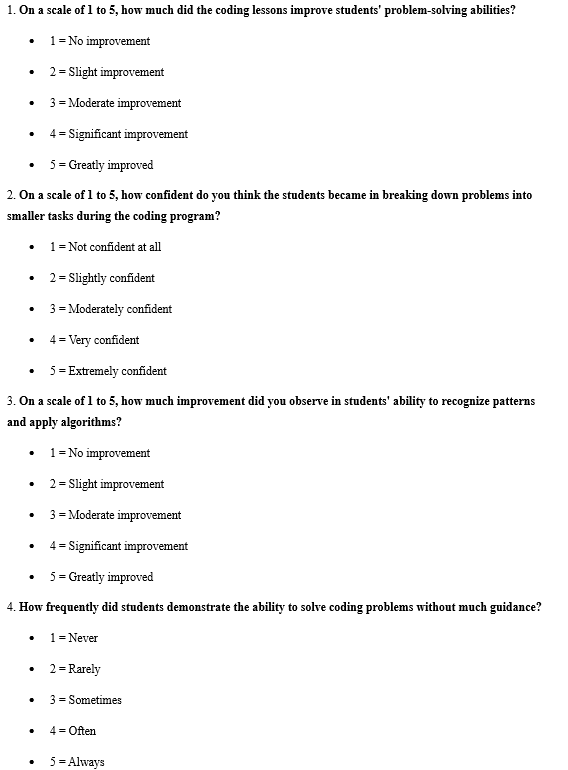


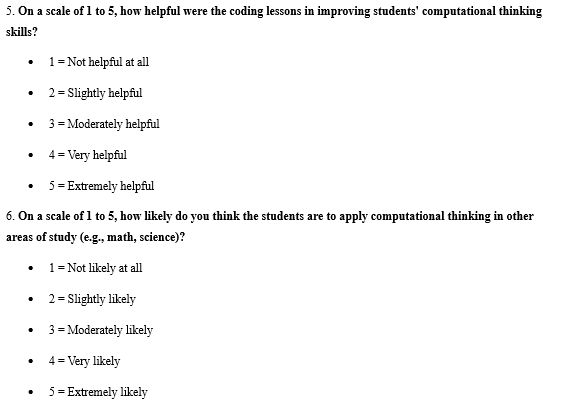

Supplement: Supplementary file 1 [file Supplementary_file_1.docx]
